# Enhancing Ras cheese safety: antifungal effects of nisin and its nanoparticles against *Aspergillus flavus*

**DOI:** 10.1186/s12917-024-04323-1

**Published:** 2024-10-29

**Authors:** Esraa Y. Abd-Elhamed, Tawfik Abd El-Rahman El-Bassiony, Wallaa M. Elsherif, Eman M. Shaker

**Affiliations:** 1 Department of Food Hygiene, Faculty of Veterinary Medicine, Sohag University, Sohag, Egypt; 2 Department of Food Hygiene, Faculty of Veterinary Medicine, Assuit University, Assuit, Egypt; 3 Nanotechnology Research and Synthesis Unit, Animal Health Research Institute, Agriculture Research Center & Faculty of Health Sciences Technology, Assiut, Egypt

**Keywords:** Ras cheese, Nisin, Nanoparticles, *Aspergillus flavus*, Food preservative, Cytotoxicity

## Abstract

**Background:**

Due to the adverse effects of industrial chemicals and their carcinogenicity and toxicity for humans, the debates have increased on using natural preservatives. This study was conducted to investigate the inhibitory effect of pure nisin and nisin nanoparticles (nisin NPs) against *Aspergillus flavus* in vivo by inoculation in laboratory-manufactured Ras cheese. A novel, safe, and natural approach of nanoprecipitation using acetic acid was employed to prepare nisin nanoparticles. The prepared NPs were characterized using zeta-sizer, FTIR, and transmission electron microscopy (TEM). Furthermore, the cytotoxicity of nisin NPs on Vero cells was assessed. The minimum inhibitory concentrations (MICs) of nisin and its nanoparticles were determined in vitro against *A. flavus* isolates using the agar well-diffusion method. The sensory evaluation of manufactured Ras cheese was conducted over a 60-day storage period.

**Results:**

The obtained results showed a strong antifungal activity of nisin NPs (0.0625 mg/mL) against *A. flavus* strain in comparison with pure nisin (0.5 mg/mL). Notably, the count decreased gradually by time from 2 × 10^8^ at zero time and could not be detected at the 7th week. The count with pure nisin decreased from 2 × 10^8^ at zero time and could not be detected at the 10th week where it’s enough time to produce aflatoxins in cheese. The MICs of nisin and nisin NPs were 0.25 and 0.0313 mg/mL, respectively. Nisin NPs used in our experiment had good biocompatibility and safety for food preservation. Additionally, the sensory parameters of the manufactured Ras cheese inoculated with nisin and nisin NPs were of high overall acceptability (OAA).

**Conclusions:**

Overall, the results of this study suggested that adding more concentration (˃0.0625 mg/mL) from nisin nanoparticles during the production of Ras cheese may be a helpful strategy for food preservation against *A. flavus* in the dairy industry.

**Supplementary Information:**

The online version contains supplementary material available at10.1186/s12917-024-04323-1.

## Introduction

Ras cheese is one of the main traditional hard cheeses; it is typically made from raw cow’s milk or a mixture of raw cow’s and buffalo’s milk. It is marketed when it develops a sharp, pungent flavor after three to six months. However, it can occasionally be stored in humid, unhygienic conditions that promote the growth of yeast and molds. Mold growth in cheese poses a serious risk to the safety and quality of food, in addition to substantial economic losses [1]. Fungi play a crucial role in spoiling food during storage as they can grow in environments where other bacteria cannot, and on a variety of substrates. This reduces the nutritional value of the food and can lead to the production of mycotoxins, which are extremely harmful to human health and make the food unsafe for consumption [2].

Nisin is a bacteriocin, specifically a naturally occurring water-soluble antimicrobial peptide (AMP) produced by the *Lactococcus lactis* bacteria. It is composed of 34 amino acid residues [3]. Due to its ability to suppress hazardous foodborne microorganisms, it is widely used as a food preservative in dairy and meat products [4]. The commercial form of nisin has been authorized as a safe food preservative by the World Health Organization, the Food and Agriculture Organization, and the US Food and Drug Administration (FDA) in 1988 [5, 6]. According to the aforementioned permissions, it is used extensively in the food industry across over 50 countries worldwide as a safe and natural food preservative that doesn’t interfere with the normal functioning of the gastrointestinal system or food [7].

Thanks to its high level of activity against gram-positive bacteria, fungi, protozoa, and tumor cells, nisin is also considered a natural antioxidant agent, nontoxic, flavorless, heat stable, and tolerant of low pH [8, 9]. Furthermore, nisin is not harmful to humans and leaves no residue because it is enzymatically broken down by human proteases [10]. Nisin, for example, was added to several varieties of cheese [11–13] skim milk, and whole milk as a bio-preserving component [14–16].

The potential of free nisin to interact with several food components, including proteins, phospholipids, fatty acids, and proteolytic enzymes, as well as its high pH and numerous other food additives, could hinder its antimicrobial efficacy as a food bio-preservative. The antimicrobial action of nisin may be greatly reduced or eliminated by these components [17]. Various techniques were developed to improve the preservation efficacy of nisin, such as liposomes [18] and nanoparticles [19].

However, these techniques are not suitable for use in the food industry because they involve the use of chemical compounds and inorganic solvents, in addition to being expensive and complicated. These reasons led to the development of environmentally friendly organic compounds, solvents, or synthetic nanoparticles that shield and release free nisin gradually, preventing it from being inactivated by a variety of food products [20]. For example, acetic acid is a well-known biocompatible organic acid that can be used as a food additive without causing adverse effects or dietary restrictions. It is a natural preservative that is frequently used to preserve food, especially cheese and dairy products, as it prevents the growth of bacteria, yeast, and fungi [21, 22].

There has been a saying for a long time “Necessity is the mother of invention”. So, nanotechnology, by the year 2000, was universally recognized as a landmark innovation, and named the sixth truly revolutionary technology introduced in the modern world and become an emerging technology for food preservation [23].

No doubt that nanoparticle systems have the potential to increase food shelf life, improve food safety and security, and minimize food spoilage [24, 25]. Furthermore, nanoparticles (NPs) were generally associated with increased catalytic behavior, chemical activity, and biological activity in comparison to big particles of the same composition. NPs are added to food as flavorings, preservatives, antibacterial sensors, and packaging materials [26].

Nano-techniques can protect nisin as a bio-preservative against many adverse conditions and efficiently prevent microbial growth and food spoilage [27]. To date, different nano-approaches have been proposed to increase the biological efficiency of nisin.

The current study aimed to prepare nisin NPs using a simple nanoprecipitation technique with natural, safe, and biocompatible materials. Also, the objectives of this study were extended to investigate the antifungal effect of obtained nanoparticles *on A. flavus* during the manufacturing and storage of Ras cheese. Additionally, the effect of the used nisin NPs on the organoleptic properties of Ras cheese was discussed.

## Materials and methods

### Preparation of Nisin nanoparticles

Nisin nanoparticles were prepared at the Nanotechnology Research and Synthesis unit, Animal Health Research Institute, Assiut Branch. Nisin (obtained from Med CHEM Express Company, India) at 2 mg/mL was fully dissolved in 100 mL of 0.1 M aqueous acetic acid solution. Then a certain amount (50 mL) of deionized distilled water was added dropwise to the nisin solution while keeping the pH value within the range of 5 to 5.5. The dispersion was constantly stirred at 30℃ for 5 h. Finally, the nanoparticle suspensions were then sonicated using a 700 kHz ultrasonic Homogenizer (BioLogics Nano-Q700, USA, Warminster, Pennsylvania) at 25 °C for 10 min before being stored in a refrigerator for further use [28].

### Characterization of the prepared nisin NPs

#### Measurement of particle size and polydispersity index (PDI)

The particle size and PDI of nanoparticles were measured at 25 ± 0.2 °C by Zeta sizer (3000 HS, Malvern Instruments, Malvern, UK) in the Faculty of Pharmacy, Al-Azhar University, Assiut. For measuring particle size, weighed amount of formulations were dispersed in double-distilled water (1:20) to avoid multiple scattering effects and obtain homogenous dispersion. This mixture was to be utilized immediately for both PDI and particle size measurement [29]. The PDI can range from 0 to 1, where 0 (zero) stands for a monodisperse system and 1 for a poly-dispersed particle dispersion.

#### Fourier-transform infrared spectroscopy (FTIR) spectral analysis

FTIR was performed in the analytical chemistry accredited laboratory, Chemistry Department, Faculty of Science, Assiut University, Egypt. It is used for identifying the functional groups with their means of attachment and the fingerprint of the molecule. For performing FTIR, samples were prepared by employing suitable methods such as the potassium bromide pellet method, and Nujol mulls and then samples were scanned in FTIR spectrometer (The FLUOstar Omega microplate reader is designed and manufactured by BMG LABTECH in Germany) in the wave number range of 4000 –500 cm^− 1^ [30].

#### High-resolution transmission electron microscope (HRTEM)

The morphology of the prepared nisin NPs was determined using HRTEM, this part was carried out in the Electronic Microscope Unit, Assiut University. Transmission electron microscopy (TEM) is used for studying the morphology of NPs through negative-staining electrons. For performing TEM, a few drops of nisin NPs were diluted with deionized water and filtered through a filter measured 200 nm then samples were placed onto nanocarbon-coated copper grids for 1 min. And negatively stained with phosphor tungstic acid for 10 min at room temperature. Later, the grids were observed in a transmission electron microscope (PHILIPS, model CM10) at an acceleration voltage of 100 kV [31].

#### Assessment of the prepared nisin NPs cytotoxicity

The cytotoxicity of the nisin NPs was evaluated using the SRB assay against a green monkey kidney Vero cell obtained from Nawah Scientific Inc., (Mokatam, Cairo, Egypt). The cells were kept in a humidified environment with 5% (v/v) CO_2_ at 37 °C, and maintained in Dulbecco’s Modified Eagle’s Medium “DMEM” supplemented with 10% heat-inactivated fetal bovine serum, 100 mg/mL streptomycin, and 100 units/mL penicillin, according to Skehan et al. [32]

Aliquots of 100 µL cell suspension (5 × 10^^3^cells) were seeded in 96-well plates and incubated in complete media for 24 h. Cells were treated with another aliquot of 100 µL of media-containing drugs at various concentrations. Following a 72-hour drug exposure period, cells were fixed by replacing media with 150 µL of 10% TCA “Trichloroacetic acid” and incubated at 4 °C for 1 h. After removing the TCA solution, and the cells were washed 5 times with distilled water. Aliquots of 70 µL SRB solution (0.4%w/v) were added and incubated at room temperature for 10 min in a dark environment. Plates were washed 3 times with 1% acetic acid and allowed to air-dry overnight. After that, 150 µL of TRIS (10 mM) was added to dissolve the protein-bound SRB stain, and a BMG LABTECH^®^-FLUO star Omega microplate reader was used to detect the absorbance at 540 nm [33]. Every experiment was carried out three times.

### Inoculum preparation

The tested mold (*A. flavus* strain) was previously isolated from Ras cheese samples by the culture method and confirmed by morphological and microscopical examination at Assuit University Moubasher Mycological Center (AUMMC) [34], (see Additional file 1). The organism was reinoculated in Sabaroud Dextrose Agar (SDA) (HiMedia, India) plate for reactivation and incubated at 25 °C for 5 days. According to Peláez et al. [35] fungal suspension was prepared with a concentration of 0.5 McFarland standard (1.5 × 10^8^ CFU/mL), where loopful from fungus was serially diluted in 0.1% sterile saline solution and compared to standard to obtain the desired inoculum level 10^8^ CFU/mL. Next, the suspension was diluted to a concentration of (1.5 × 10^7^ CFU/mL) by diluting 1 mL of the suspension (0.5 McFarland standard) in 9 mL of sterile saline 0.1%. This resulted in a concentration of (1.5 × 10^7^ CFU/mL) [36].

### Determination of the minimum inhibitory concentration (MIC) of free nisin and nisin nanoparticles against *A. flavus* strain

To determine the MIC of nisin and nisin NPs against *A. flavus* strain, the agar well diffusion method was used, where 100 µL of the previously prepared *A. flavus* suspension containing 1.5 × 10^7^ colony forming unit (CFU/mL) were spread on Muller Hinton agar plates using a sterile L shape glass rod and left for 10 min to be absorbed. Then, 4 mm diameter wells were punched into the agar plates using a sterile cork borer to test the antifungal activity of nisin and nisin NPs. A two-fold serial dilution of nisin and nisin NPs was prepared in deionized water to achieve a decreasing concentration range from 2 to 0.0078 mg/mL. 100µL of different concentrations of free nisin and nisin NPs (from 2 to 0.0078 mg/mL) were poured into wells. 100 µL of sterile deionized water was preserved in one well for each plate and kept as a negative control. The plates were incubated at 25 °C for 5 days. Then, the diameters of the inhibition zones were measured in mm to determine the MIC [37].

### The antifungal efficacy of free nisin and nisin NPs against the *A. flavus* isolates inoculated in manufactured Ras cheese during ripening period

Ras cheese was laboratory manufactured according to Hammam et al. [38]. Three groups were inoculated with the inoculum, one without treatment i.e. containing *A flavus* strain only, and two other groups were inoculated with *A flavus* strain and treated with double MIC of nisin and nisin NPs. Moreover, the same three groups but without inoculum were kept as a negative control for organoleptic examination. Then the cheese is pressed overnight and salted on both sides with dry salt for 2 weeks. After that, the cheese was packaged in sterile polyethylene bags and stored at 4 °C. Ten g from each positive group of Ras cheese were aseptically homogenized with 90 mL sterile 2% sodium citrate solution in a homogenizer (HG-15D, DAIHAN Scientific Co., Ltd., Korea, 230 V, 50/60 HZ, watts 160 W) to obtain 1/10 dilution, followed by tenfold serial dilutions using a vortex to homogenate the obtained solution. Take 0.1 mL from each dilution and spread over a dry surface of Sabaroud Dextrose Agar (SDA) (HiMedia, India) plates containing chloramphenicol and vancomycin (0.05 g/L), using a bent glass rod till complete absorption of the inoculum. The inoculated plates were incubated in an inverted position in a cooled incubator at 25 °C for 5–7 days. *A. flavus* was counted at zero time, after 24 h., 48 h., and every week until obtained negative results or deterioration of stored cheese.

### Sensory evaluation of Ras cheese during the storage period

The sensory evaluation of the three negative groups of Ras cheese over a 60-day storage period was examined by four key attributes: flavor, body & texture (firmness), appearance & color, and overall acceptability (OAA) and were compared. The level of agreement was scored [39].

### Statistical analysis

SPSS program version 14 (SPSS Inc., Chicago, IL, USA) was used to detect the mean and standard deviation of nisin NPs cytotoxicity.

## Results

### Characterization of the prepared nanoparticles

The average dynamic droplet size of nisin NPs and the PDI measurements by using a zeta sizer. The PDI of nisin NPs was 0.127 with an average droplet size of 55.74 ± 6.7 nm (Table 1**).**

**Table 1:** Physical properties of the prepared nisin NPs

Type of nano-materials	Average droplet size (nm)	Polydispersity index (PDI)
Nsin NPs	55.74 ± 6.7	0.127

These findings indicated that acceptable small-sized particles of nisin were obtained by precipitation technique using acetic acid. The produced nisin NPs showed good stability and a mono-size dispersion, as evidenced by their small size and narrow PDI measurements.

**Fig. 1:**
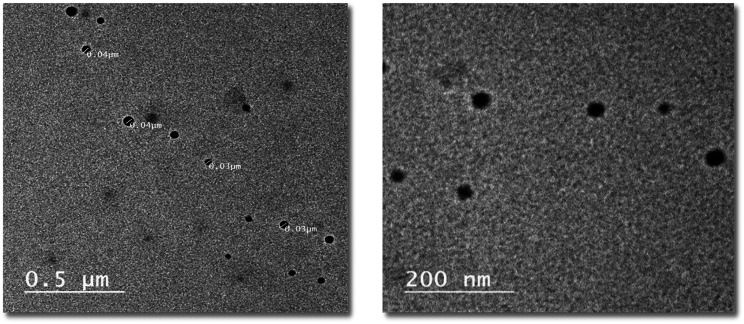
Morphological study of nisin NPs by using Transmission Electron Microscope with average size 35 ±5.78 nm

The morphology and size of the prepared nisin NPs fabricated by nanoprecipitation were determined by HRTEM and are presented in Fig. 1 where separate spherical shape particles with an average size of 35 ± 5.78 nm without any aggregation and perfect stability. The image revealed that most of the nanoparticles were almost spherical, which provides more antifungal activity. They also had an approximately uniform shape and distribution in the solution. Moreover, the TEM image revealed that the nanoparticles were evenly distributed in the aqueous phase. Because TEM determines the actual particle diameter and the zeta-sizer determines the particle diameter with adjacent moving layers of liquids, the size of particles measured by TEM is typically lower than the dynamic particles estimated by the zeta-sizer.

**Fig. 2:**
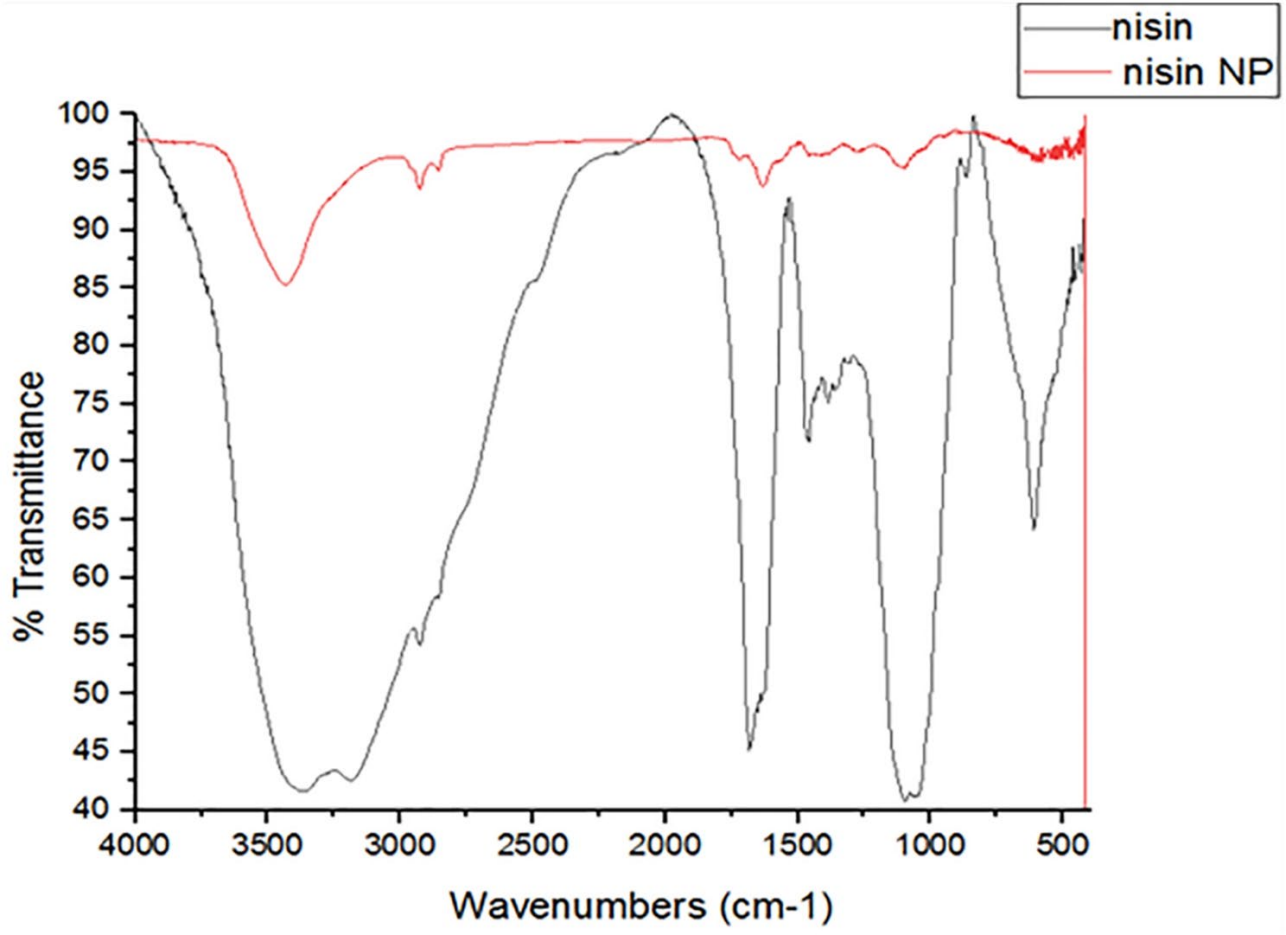
FTIR of nisin and its nanoparticles

The FTIR spectra of pure nisin and nisin nanoparticles were displayed in Fig. 2. The nisin spectrum displayed distinct peaks at 3425, 1599, and 1493 cm^− 1^, which were associated with O–H stretching of COOH, C = O stretching of amide I, and N–H bending of amide II. The stretching of amid II was shown by bands 1530 cm^− 1^ in free nisin, which increased to 1549 cm^− 1^ in nisin NPs, indicating an increase in the H-bond in nanoform compared to free one. The FTIR spectrum data verified that there were no chemical alterations or interactions between nisin and the materials employed in the synthesis of nisin nanoparticles. These outcomes also showed that the technique used was appropriate for producing small-sized, chemically stable nisin NPs.

### Assessment of Nisin nanoparticles cytotoxicity

**Fig. 3:**
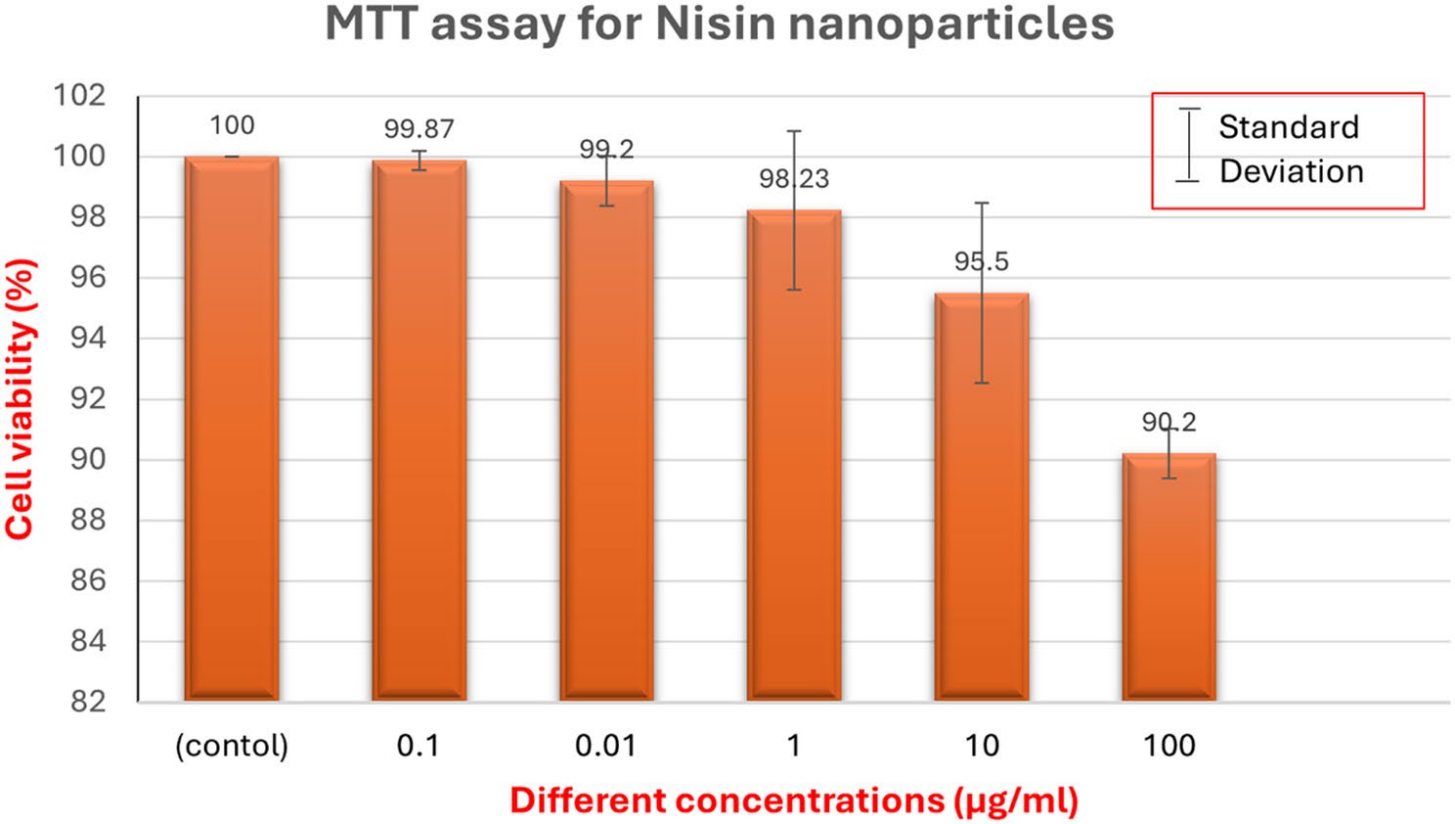
Cytotoxicity of Nisin nanoparticles

Nisin NPs were applied to Vero cells for 24 h, and MTT assays were used to gauge the cytotoxicity. The Vero cell did not show any anti-proliferative effect from the MIC, according to the results in Fig. 3. Interestingly, 92% of viable cells remained after 24 h., indicating that nisin NPs did not cause cytotoxicity even at very high concentrations. These findings confirmed that the MIC of nanomaterials used in our experiment had good biocompatibility and safety for food preservation.

### MIC of free nisin and nisin NPs against *A. flavus* strain

Using the agar well diffusion method, the effectiveness of free nisin and prepared nisin NPs was investigated (Table 2). Nisin and its nanoparticles showed a potent antifungal effect against *A. flavus.* When throw the light on Table 2, the MICs of nisin and nisin NPs were 0.25 and 0.0313 mg/mL, with growth inhibition zones of 13 and 11 for pure nisin and nisin NPs, respectively.

**Table 2:** MIC of nisin and nisin nanoparticles by mm using agar well diffusion method

Antifungal Substances	Concentrations (mg/mL)	The diameter (mm)
Pure nisin	2 1 0.5 0.25 0.125 0.0625 0.0313 0.0156 0.0078	21 17.5 14 13 NZ NZ NZ NZ NZ
Nisin NPs	2 1 0.5 0.25 0.125 0.0625 0.0313 0.0156 0.0078	23 19 17 14 14 12 11 NZ NZ

NZ: No Zone

### The antifungal efficacy of free nisin and nisin NPs against the *A. flavus* isolates inoculated in manufactured Ras cheese

The applied in-vivo study reflected the effectiveness of the used nisin and its nanoparticles against the inoculated *A. flavus* strains. From the results recorded in Table 3, the nisin NPs were more effective than free nisin against *A. flavus* strains, in which the count decreased from 2 × 10^8^ at zero time to 10 at the 6th week. While the count with free nisin decreased from 2 × 10^8^ at zero time to 100 at the 9th week. The count in positive control began to decrease after 2 months, which may be due to dryness which is due to the small sample size. Also, the results obtained in this table showed that the count reached zero with a reduction percent 100% after the 6th week in nisin NPs and the 9th week in nisin.

**Table 3:** Antifungal effect of Nisin and its nanoparticles on *Aspergillus flavus* growth in laboratory manufactured Ras cheese

Storage period	Positive control	Treated with Nisin (0.5 mg/mL)		Treated with Nisin nanoparticles (0.0625 mg/mL)	
	Count	Count	Reduction%	count	Reduction%
Zero time 24 h 48 h 1st week 2nd week 3rd week 4th week 5th week 6th week 7th week 8th week 9th week 1th week	2 × 10^8^ 2 × 10^8^ 3 × 10^8^ 1 × 10^9^ 6 × 10^9^ 1 × 10^10^ 3 × 10^10^ 5 × 10^10^ 5 × 10^10^ 6 × 10^10^ 4 × 10^9^ 1 × 10^9^ 6 × 10^8^	2 × 10^8^ 3 × 10^7^ 1 × 10^6^ 7 × 10^5^ 1 × 10^5^ 6 × 10^4^ 8 × 10^3^ 2 × 10^3^ 8 × 10^2^ 3 × 10^2^ 9 × 10 2 × 10 ND	ـــــــــــ 85.000000 98.000000 99.000000 99.950000 99.970000 99.996000 99.999000 99.999600 99.999850 99.999955 99.99999 100	2 × 10^8^ 1 × 10^6^ 2 × 10^5^ 5 × 10^4^ 1 × 10^4^ 1 × 10^3^ 2 × 10^2^ 7 × 10 1 × 10 ND ND ND ND	ـــــــــــ 99.500000 99.900000 99.975000 99.995000 99.999500 99.999900 99.999965 99.999995 100 100 100 100

ND: Not Detected

### Sensory evaluation of the laboratory-manufactured Ras cheese

Table 4 clarified that the flavor score of negative control treatment improved from 7.9 at 30 days to 9.2 at 60 days, whereas the flavor score in nisin (0.5 mg/mL) increased from 8 to 8.9. In contrast, the flavor score increased from 8.1 to 9 in nisin NPs (0.0625 mg/mL). Scores of body & textures (firmness) in negative control treatment improved from 8.7 at 30 days to 9.4 at 60 days. While in nisin (0.5 mg/mL) and nisin NPs (0.0625 mg/mL) increased from 8.2 to 8.9 and 8.6 to 9.2, respectively. Appearance & Color´s scores in negative control treatment remained high, with a slight decrease from 9.7 at 30 days to 9.5 at 60 days, while in nisin (0.5 mg/mL) and nisin NPs (0.0625 mg/mL), scores slightly decreased from 9.3 to 9.1 and 9.4 to 9.1, respectively. As for overall acceptability (OAA) in negative control treatment improved from 8.7 at 30 days to 9.4 at 60 days, while in nisin (0.5 mg/mL) and nisin NPs (0.0625 mg/mL) increased from 8.5 to 9 and 9 to 9.1.

**Table 4:** Sensory evaluation of Ras cheese during the storage period

Treatment	Flavor		Body & texture (firmness)		Appearance &Color		Overall acceptability (OAA)	
	30 days	60 days	30 days	60 days	30 days	60 days	30 days	60 days
Negative control	7.9	9.2	8.7	9.4	9.7	9.5	8.7	9.4
Nisin (0.5 mg/mL)	8	8.9	8.2	8.9	9.3	9.1	8.5	9
Nisin NPs (0.0625 mg/mL)	8.1	9	8.6	9.2	9.4	9.1	9	9.1

Average score of sensory evaluation

## Discussion

For the first time, the present study clarified the mechanism by which free nisin and nisin NPs suppress *A. flavus*, one of the most dangerous foodborne pathogens, during the manufacturing of Ras cheese. At various stages of their production chain, it releases the carcinogenic mycotoxin known as “aflatoxin.” The existence of this fungus and its aflatoxins is critical to the safety of food [40]. Nisin NPs, in contrast to free nisin, have the potential to provide a significantly higher antifungal effect on *A. flavus* with a high level of customer acceptability. Consequently, nisin NPs have the potential to be an effective and beneficial bio-preservative choice in the dairy industry, counteracting *A. flavus*.

According to the current study, nisin NPs were prepared by a novel and safe method using natural material like acetic acid which is commonly used in food products. While Baboota et al. [29] prepared nisin NPs in ultra-small sizes using the nanoprecipitation method with HCL, we prepared nisin NPs with significantly lower particle sizes using acetic acid, which is safer, more consumer-accepted, and less harmful. The solvent is removed from the nisin nanoparticle samples before they are dried for TEM analysis, which may cause the nanoparticles to shrink. This could explain why the particle size obtained by the Zeta-sizer is larger than the size measured by TEM. Moreover, DLS calculates the hydrodynamic diameter of the dispersed moving particles concerning the surrounding moving layers of solvents. The results of the PDI test showed that the prepared nisin NPs were stable and had good homogeneity, with a value lower than 0.5. The precipitation method prevented the NPs from coalescing at room temperature and over an extended storage period. Furthermore, since PDI measures the homogeneity of droplet size in a nanomaterial, a higher score indicated a lesser uniformity of droplet size [41]. Additionally, samples with a PDI value greater than 0.7 and a highly wide size distribution are unsuitable for Dynamic Light Scattering (DLS) investigation [42]. As that mentioned in several previous studies [43–47]. Higher results were obtained by Ghosh et al. [48] with particle size ranging (254 –96 nm) and PDI (0.25 − 0.21).

The FTIR result agreed with the findings of Flynn et al. [49]. Here, we found that the -OH stretching peak of nisin NPs was more intense than that of free nisin, indicating a stronger creation of hydrogen bonds within nisin NPs. The peak at 1620 cm − 1 corresponding to COO − in the case of free nisin was displaced to 1610 cm − 1 in nisin NPs, indicating a rise in hydrogen bonding inside the nisin NPs. In contrast, the amide II band in free nisin appeared at 1530 cm^− 1^ and became more noticeable at 1549 cm^− 1^ in nisin NPs which agreed with Webber et al. [50]. The band of amide I at wave number 1632 cm^− 1^ may have resulted from a structural alteration of free nisin upon conversion into nisin NPs via the use of natural acetic acid and that revealed the efficacy of the antifungal activity of the prepared nano-nisin. N-H bending of amide II, C (double bond, length as m-dash) O stretching of COOH, and 3287.70, 1651.24, and 1531.68 cm^− 1^, respectively, were responsible for the distinctive peaks of nisin. The N-P-NPs’ spectra showed that the characteristic peaks for primary amines (amide II) at 1531.68 cm-1 and COO at 1651.24 cm-1 had been shifted to 1592 cm-1 and 1642 cm-1, respectively.

Nisin has been approved for use in the food chain and is frequently utilized in the food industry in more than 50 countries worldwide as a natural and safe food preservative that doesn’t interfere with digestive system functioning or food flavor [7] and was granted generally recognized as safe (GRAS) status by the FDA in 1988 [6]. Interestingly, the US FDA and FAO/WHO Codex Committee allow nisin to be added to dairy products at up to 250 mg/kg [51, 52]. Furthermore, the European Food Safety Authority states that nisin is acceptable to use as a food preservative for dairy and meat products because it is non-toxic to humans [53]. The examined mold (*A. flavus*) and its aflatoxins have been involved in many past food outbreaks worldwide due to the consumption of aflatoxins over 1–3 weeks, an AFB1 dose of 20–120 µg/kg body weight (bw) per day is acutely toxic and potentially lethal [54]. Therefore, employing either nisin or nisin NPs as natural food preservatives makes the current study a useful alternative strategy to limit the potential health risks of *A. flavus* and its aflatoxins after consumption of Ras cheese.

Nisin mainly demonstrates strong antibacterial activity through electrostatic interactions with components of bacterial cell walls, including lipid II, teichoic acid, and polysaccharides. According to Bauer and Dicks [55], this interaction results in the creation of persistent toroidal holes, which impede the manufacture of cell walls and ultimately cause cell death. These findings suggest that nisin’s antifungal action might also be dependent on damaging fungal cell walls and interfering with the conversion of yeast cells into hyphal forms [56]. The obtained results showed that nisin NPs had a lower minimum inhibitory concentration (MIC) )0.0313 mg/mL ( than pure nisin (0.25 mg/mL). This could be because nisin NPs generally exhibited higher biological, chemical, and catalytic activity when compared to large particles of pure nisin. In this work, nisin NPs were found to be more effective than free nisin at inhibiting the growth of *A. flavus* throughout the manufacture and storage of Ras cheese, as determined by counting the mold’s growth in the laboratory-made cheese. Nisin NPs with high specific surface area have the potential to bind to target cell surfaces with ease, increasing cell membrane permeability and ultimately leading to fungal cell death. Additionally, the internal non-covalent interactions within the nanoparticles gave nisin NPs their thermo-tolerance [57]. In our study, nisin NPs completely inhibited *A. flavus* after the 6th week of Ras cheese manufacture, on the contrary, the pure nisin completely inhibited *A. flavus* after the 9th week, or before the end of maturation and consumption. This is sufficient time for *A. flavus* to produce aflatoxins, so higher concentrations and preventive measures were required.

Given that nisin and nisin NPs have no taste or odor, the effect of adding them to Ras cheese’s OAA scores was observed, and the results were satisfactory [8, 9]. Utilizing the antimicrobial properties of nisin NPs to combat a range of spoilage and pathogenic microorganisms, this innovative non-thermal food preservative effectively eliminates microorganisms while minimizing any adverse effects on the safety, quality, nutritional value, and acceptability of dairy products.

Due to the increasing demand for food products without preservatives, natural antimicrobials are becoming more popular for their safety and effectiveness. This study aims to investigate the potential of adding nisin nanoparticles to curd for manufacturing Ras cheese as a novel method to reduce the risk of foodborne pathogen contamination. Further research is needed to determine the safe and effective dosage of nisin nanoparticles for use in different dairy products.

## Conclusion

This study demonstrated the efficacy of nisin and nisin NPs as natural preservatives in Ras cheese against the fungal pathogen *A. flavus*. The nisin nanoparticles (NPs) showed significantly stronger antifungal activity compared to pure nisin. They reduced the *A. flavus* count to undetectable levels by the 7th week of storage, whereas pure nisin achieved this by the 10th week. The cytotoxicity evaluation showed that nisin NPs are safe for use in food preservation. The results of the sensory evaluation indicated that the addition of nisin and nisin NPs did not adversely affect the flavor, body & texture, appearance & color, or overall acceptability of Ras cheese. The use of Nisin NPs resulted in a slight improvement in these characteristics compared to pure nisin, especially in terms of flavor and texture. Therefore, this study could be beneficial for Ras cheese factories by increasing the concentration of Nisin NPs and using them as a preventative method to inhibit the growth of *A. flavus* in Ras cheese and subsequently, avoid food spoilage and foodborne diseases. It is suggested that nisin nanoparticles be applied to more food categories, particularly those that are prone to fungal contamination, to evaluate their effectiveness and impact on sensory characteristics in a range of food matrices.

## Electronic supplementary material

Below is the link to the electronic supplementary material.

Supplementary Material 1

## Data Availability

In related data to this manuscript is available upon request.
