# Supplementary material for: Enhancing Ras cheese safety: antifungal effects of nisin and its nanoparticles against Aspergillus flavus
Source: BMC Vet Res. 2024 Oct 29;20:493. doi: 10.1186/s12917-024-04323-1 (PMC11520377; doi:10.1186/s12917-024-04323-1)
Supplement: Supplementary file 1 — Supplementary Material 1 [file 12917_2024_4323_MOESM1_ESM.docx]

**Additional file (1)**

| 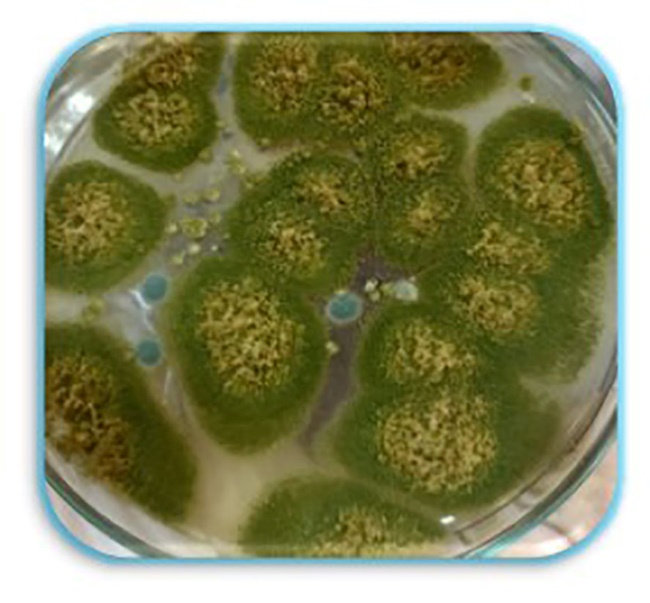 | 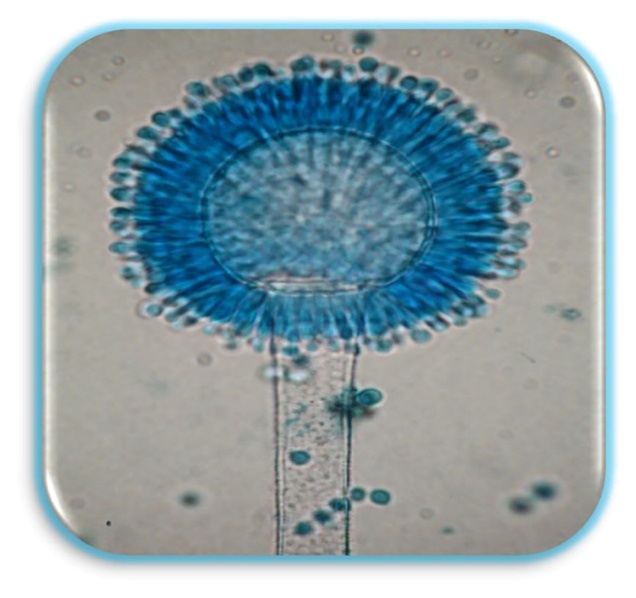 |
| --- | --- |
| Growth of *Aspergillus flavus* on Sabaroud Dextrose Agar (SDA) | Microscopical examination of *Aspergillus flavus* strains. |
